# Supplementary material for: FunHoP analysis reveals upregulation of mitochondrial genes in prostate cancer
Source: PLoS One. 2022 Oct 25;17(10):e0275621. doi: 10.1371/journal.pone.0275621 (PMC9595552; doi:10.1371/journal.pone.0275621)
Supplement: S3 File — (PDF) [file pone.0275621.s003.pdf]

Supporting information

Supporting S1-S3 Figs

The figures show different aspects of mitochondrial genes in the dataset, using the consensus data on subcellular localization. In each figure the four pathways chosen for further analysis are indicated.

S1 Fig: Mitochondrial pathways – number of genes

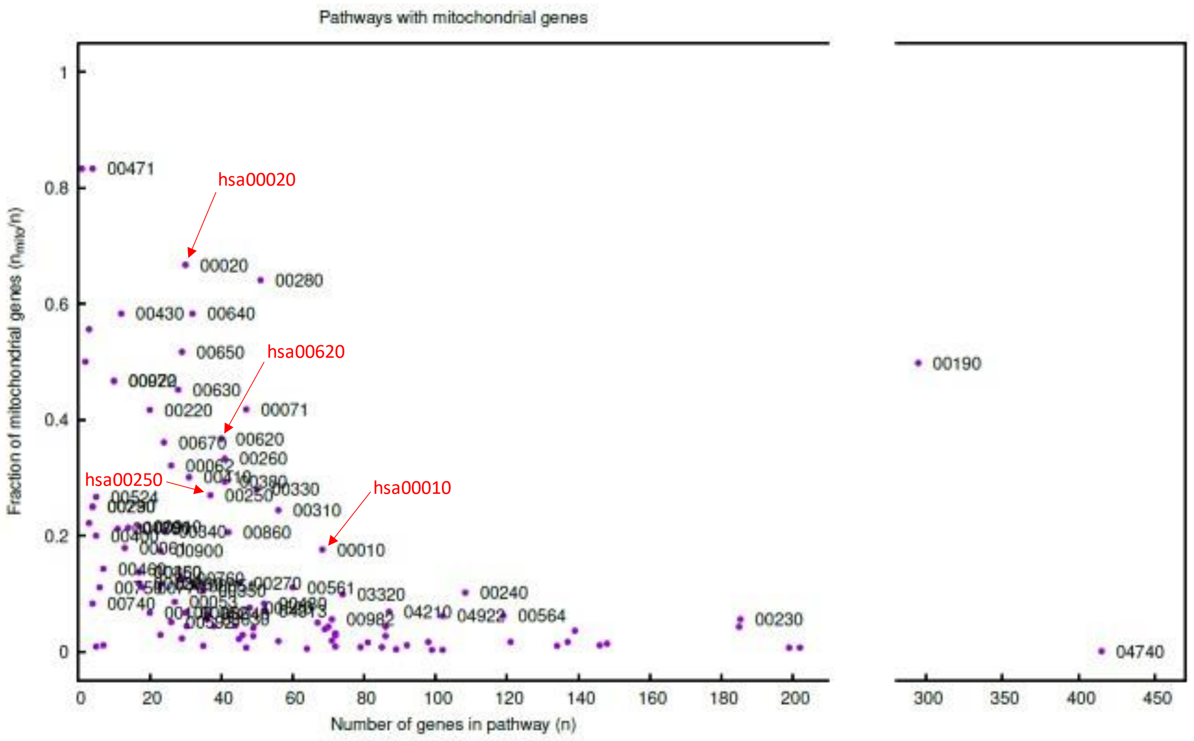

This figure shows the fraction of genes in each pathway that are mitochondrial genes, relative to the number of genes in each pathway. We can for example see that for hsa00280 just over 60% of approximately 50 genes are mitochondrial. The figure shows that the number of pathways with a high percentage of mitochondrial genes is limited.

S2 Fig: Mitochondrial pathways – number of regulated genes

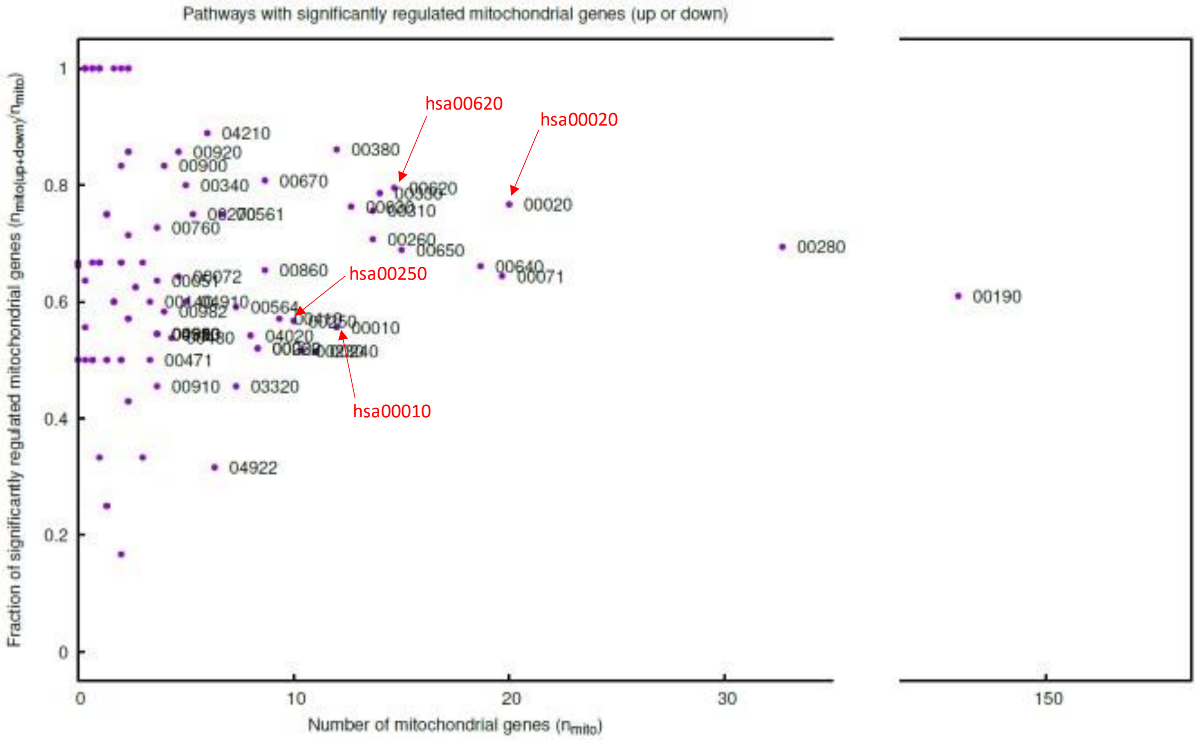

This figure shows the fraction of mitochondrial genes in each pathway that are significantly regulated, both up and down. We see for example that for has00380 more than 80% of the at least 10 mitochondrial genes are significantly regulated. The figure shows clearly that in most pathways where some mitochondrial genes are found, these genes are in general actively regulated.

S3 Fig: Mitochondrial pathways – number of upregulated genes

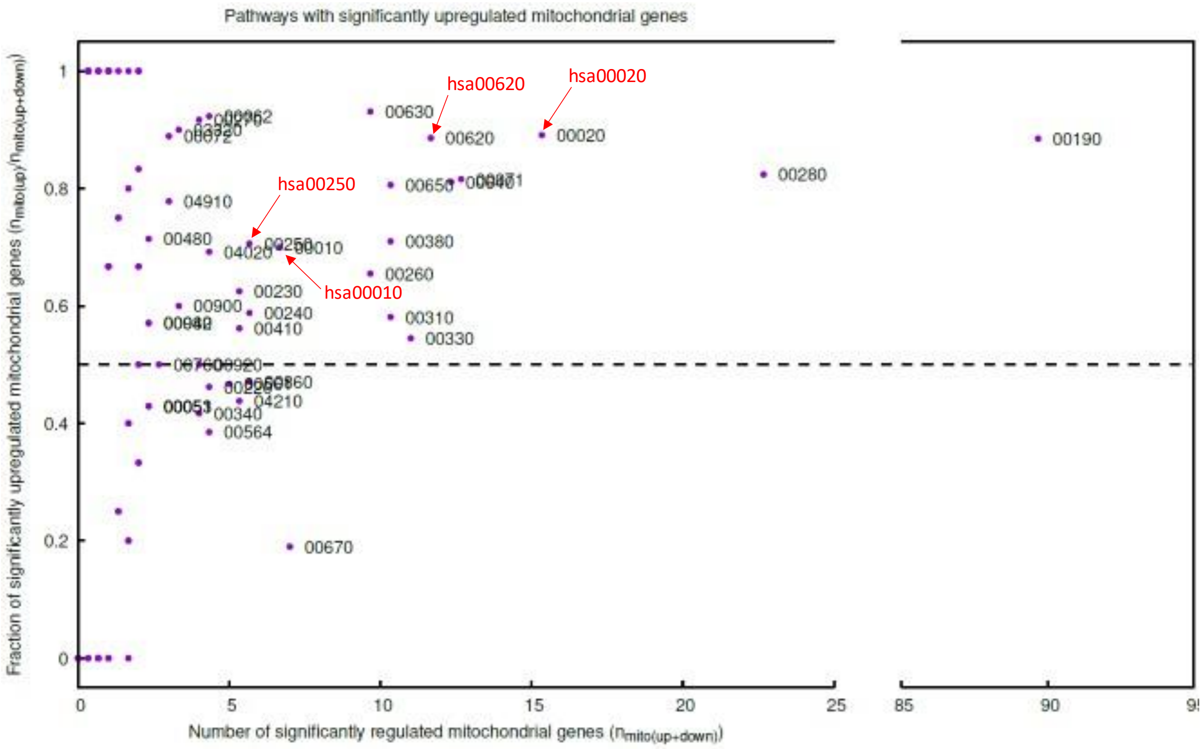

This figure shows the fraction of significant regulated mitochondrial genes that are upregulated. We can for example see that for hsa00020 approximately 90% of the around 15 actively regulated mitochondrial genes are upregulated. The figure shows clearly that upregulation of mitochondrial genes is the most common situation, and that pathways with downregulated mitochondrial genes in general has very few such genes.

Supporting S4 and S5 Figs.

These figures show the original metabolic pathways of The TCA Cycle and Glycolysis/Gluconeogenesis

S4 Fig. The TCA cycle - original version.

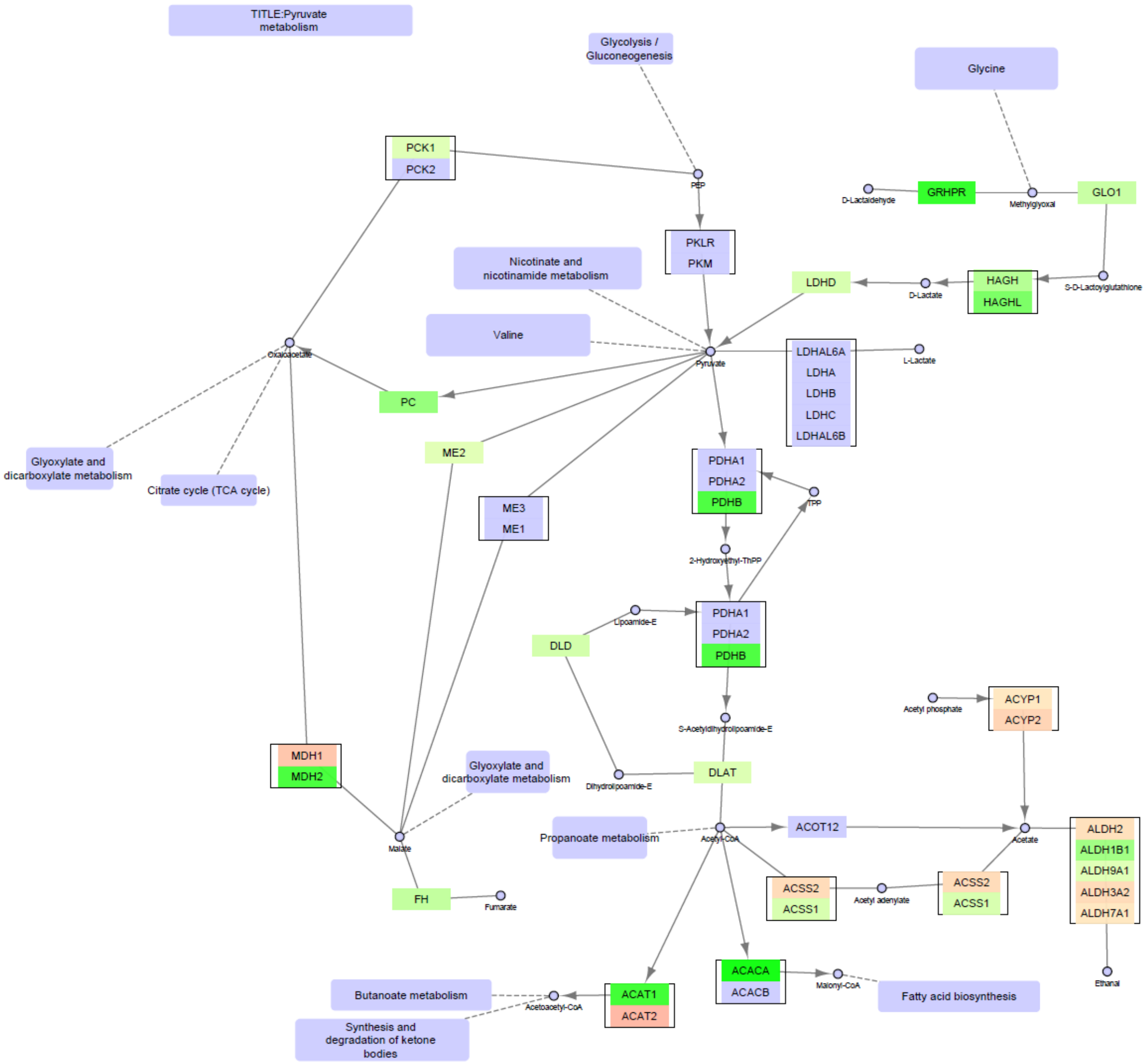

S5 Fig. Glycolysis/gluconeogenesis - original version.

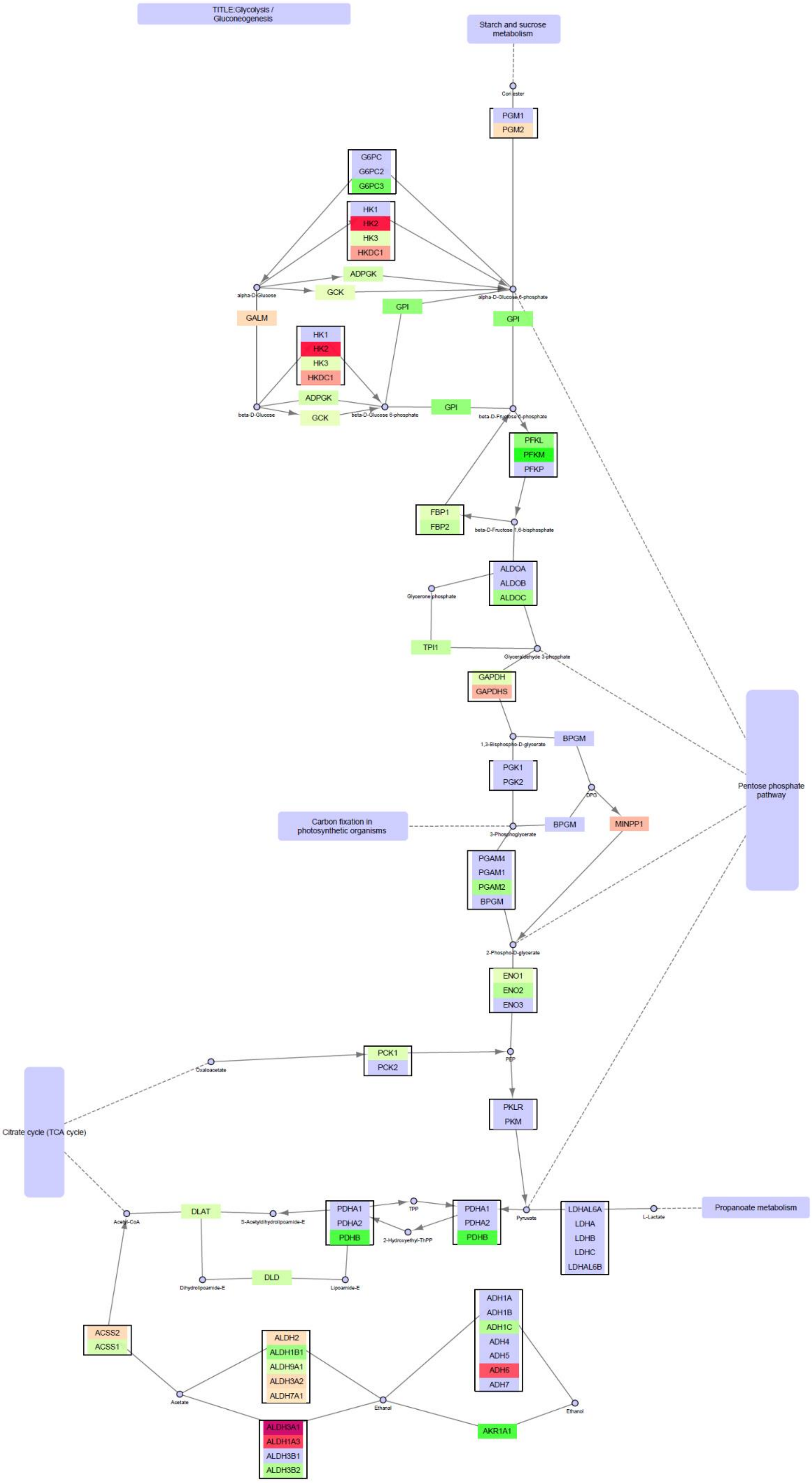

Supporting S1 and S2 Tables

S1 Table – Overlap between categories when comparing the datasets

HPA	*SubCell*						
	Uncl	Uncrt	Mito	Unkn	Secr	Nucl	Cyto
Uncrt	4	5	9	51	58	26	133
Nucl	3	17	7	73	47	282	213
Mito	4	8	141	25	15	6	25
Unkn	17	21	60	829	174	44	130
Secr	8	16	8	122	215	17	102
Cyto	5	8	8	86	45	15	259
HPA	*BUSCA*						
	Memb	Cyto	Unkn	Mito	Nucl	Extra	
Uncrt	67	130	0	24	39	26	
Nucl	66	251	2	41	265	17	
Mito	27	32	1	154	4	6	
Unkn	739	184	46	101	60	145	
Secr	231	111	2	23	37	84	
Cyto	60	244	6	33	56	27	
BUSCA	*SubCell*						
	Uncl	Uncrt	Mito	Unkn	Secr	Nucl	Cyto
Memb	16	14	20	726	334	25	55
Cyto	6	24	19	181	79	105	538
Unkn	0	2	12	27	5	3	8
Mito	4	15	172	62	24	19	80
Nucl	5	11	4	65	22	213	141
Extra	10	9	6	125	90	25	40

Mito – Mitochondria; Nucl – Nuclear / Nucleus; Cyto – Cytosol / Cytoplasm; Memb – Membrane; Extra – Extracellular; Secr – Secretory; Unkn – Unknown; Uncl – Unclassified; Uncrt - Uncertain

This table shows the overlap as number of genes between different categories when comparing HPA data to SubCell, HPA to BUSCA, and BUSCA to SubCell.

For making Fig 1 the BUSCA categories Extracellular and Membrane were combined as Secretory. The data here seems to support this, as these two categories show the largest overlap to Secretory both for HPA and SubCell (although most of the Extracellular and Membrane genes are Unknown (i.e., without classification) in the experimental data).

S2 Table – Comparison of predicted and experimental localization data

	TP	FP	FN	TN	TPR	TNR	PPV	ACC
BUSCA vs. HPA	131	78	55	1130	0.70	0.94	0.63	0.90
BUSCA vs. SubCell	127	82	36	1149	0.78	0.93	0.61	0.92
HPA vs. SubCell	141	45	22	1186	0.87	0.96	0.76	0.95
SubCell vs. HPA	141	22	45	1186	0.76	0.98	0.87	0.95

TP – True positive predictions
FP – False positive predictions
FN – False negative predictions
TN – True negative predictions
TPR – True positive rate, sensitivity – TP / (TP + FN)
TNR – True negative rate, specificity – TN / (TN + FP)
PPV – Positive predictive value, precision – TP / (TP + FP)
ACC – Accuracy – (TP + TN) / (TP + TN + FP + FN)

The purpose of this analysis has been to assess the reliability of predicting mitochondrial localization with BUSCA, using HPA or SubCell as a reference. In addition, also the experimental data have been evaluated for reliability, using data from one method as “prediction” and the other as reference. The localization data have been simplified into “mitochondrial” and “everything else”. The comparisons have been done over the set of genes that has localization data in all three datasets, in total 1394 genes. This represents 42% of the genes with gene products analyzed in the KEGG pathways in the main paper.

The analysis shows that the specificity is very high, indicating that the predictions are quite reliable when a localization has been predicted to be mitochondrial.
