# Supplementary material for: FunHoP analysis reveals upregulation of mitochondrial genes in prostate cancer
Source: PLoS One. 2022 Oct 25;17(10):e0275621. doi: 10.1371/journal.pone.0275621 (PMC9595552; doi:10.1371/journal.pone.0275621)
Supplement: Attachment — Submitted filename: Response_to_Reviewers.docx [file pone.0275621.s004.docx]

**Response to Reviewers.**

**General:**

First we would like to inform you that the previous Figure references 4a, 4b and 4c have been replaced by Figure references 4, 5 and 6 respectively, and that Figure references 5a, 5b and 5c have been replaced by Figure references 7, 8 and 9 respectively. The reason for this change was to present this as standalone figures rather than figure panels, since using figure panels would compromise the resolution and readability of all these figures considerably.

**Reviewer #1:**

3. Have the authors made all data underlying the findings in their manuscript fully available?

Include the original versions of the TCA cycle and Glycolysis/gluconeogenesis pathways associated with figures 2 and 3. They could be included in the supplemental to not detract from the presented mitochondrial/non-mitochondrial pathway. The original version of the Glycolysis/gluconeogenesis is referenced in both the results and discussion. Originals are already provided for Pyruvate metabolism and Alanine, glutamine, and glutamate metabolism pathways.

Reply: We agree with the reviewer, and we have added the original versions of TCA cycle and Glycolysis to the Supporting Information (S4 Fig and S5 Fig respectively).

4. Is the manuscript presented in an intelligible fashion and written in standard English?

Fig 5b is of lower quality than a and c. It is very difficult to read.

Reply: We have replaced Fig 5b with a new version of better quality. It is now presented as Fig 8 (see comment above)

**Reviewer #2:**

1. A notable weakness of this study is that the results were not quantified. Although most mitochondrial genes are upregulated in the TCA cycle, other pathways may show similar numbers of upregulated and downregulated genes that belong to both mitochondrial and non-mitochondrial proteins. Can the authors develop a quantitative score to measure the entire pathway (e.g., by combining fold changes of up- or down-regulated genes)?

Reply: The Supporting Information (now included with the submission) includes several plots that illustrate the distribution of mitochondrial proteins according to key properties (fraction of genes in each pathway, fraction with significant regulation, fraction with significant upregulation). We believe that these data give some of the relevant information that the reviewer asks for.

However, “to develop a quantitative score to measure the entire pathway” that aggregates fold changes, as suggested by the reviewer, is much more complicated and outside the scope of the current project.

2. It is important to provide more details in the gene expression analysis. There are two normal cell lines and two prostate cancer (PCa) cell lines. This could yield 4 different datasets. It is not clear how the authors combined them to produce only one set of results for downstream analysis. In addition, the authors should include the results in the supplementary material.

Reply: Initially wanted to look for differences in all four cell-lines. However, when using various methods to compare cells based on differential expression results, we found that the two cancer and two normal cell-lines shared the most similarities. In addition, comparing all four cell-lines against each other would have made the analysis too comprehensive. We thus decided to focus on compartmental difference in cancer vs normal metabolism by combining the cancer and normal cell-lines. The results from differential analysis is provided in Supplemental S1 File.

3. A better discussion is needed when interpreting the mitochondrial and non-mitochondrial versions of the pathway. Do they imply that these reactions take place exclusively inside or outside the mitochondria? Or does the split pathway imply that these proteins need to be transferred into (or out of) mitochondria. In this case, the non-mitochondrial version of the TCA cycle would imply that these proteins need to be transferred into the mitochondria, even though they are considered non-mitochondrial proteins.

Reply: The reviewer addresses a very relevant issue with this comment. In this study we have assumed that the metabolic enzymes have their activity in either mitochondrial or cytosolic compartments. Though import/export of these enzymes from the mitochondria takes place, we have not implemented this in our analysis. For example for mitochondria, most of the relevant proteins are made in the cytosol and must afterwards transported into the mitochondria, and changes in protein transport may affect the distribution of these proteins between cytosol and mitochondria. Moreover, we do not have data on whether this transport affects protein activity in the compartments. This is a limitation of our study, and we have added a brief comment about this to the discussion.

Metabolites themselves can move between compartments with the help of transport proteins. The role of transport proteins is not well modelled in current metabolic pathway schemes (like KEGG) but could be a natural and important expansion to such pathways. This includes both transport in and out of the cell itself, as well as transport between different compartments within the cell. It is clear that such transport can greatly affect the balance and availability of metabolites in the different compartments, and lack of modelling of metabolite transport is a clear limitation in metabolic pathway analysis.

We have added a short section on compartments and transport of metabolites between compartments to the introduction, and also elaborated on issue in the discussion.

4. Related to point 3, if the idea of "transfer" is correct, then caution may be needed when integrating changes in gene expression. Even if the level of gene expression is not increased, their activity may still be increased if the level of the transfer protein is increased. Can the authors provide evidence to support this idea by examining the datasets?

Reply: As discussed above, we assume that transport that is important to our system is limited to metabolites and does not include proteins. Changes in protein transport may certainly change the picture, as indicated by the reviewer. However, we do not have access to any data that makes it possible to take this into account. We have added a short comment about this to the Discussion.

5. There is little discussion of comparing the results obtained in this study with similar studies in the literature.

Reply: We have tried to include more comparisons of our results to other studies, in particular with respect to the general increased activity of mitochondrial processes in cancers. We have added a section on this to the Discussion.
